# Activity-dependent translation dynamically alters the proteome of the perisynaptic astrocyte process

**DOI:** 10.1016/j.celrep.2022.111474

**Published:** 2022-10-18

**Authors:** Darshan Sapkota, Mandy S.J. Kater, Kristina Sakers, Kayla R. Nygaard, Yating Liu, Sarah K. Koester, Stuart B. Fass, Allison M. Lake, Rohan Khazanchi, Rana R. Khankan, Mitchell C. Krawczyk, August B. Smit, Susan E. Maloney, Mark H.G. Verheijen, Ye Zhang, Joseph D. Dougherty

**Affiliations:** 1 Department of Psychiatry, Washington University School of Medicine, St. Louis, MO, USA; 2 Department of Genetics, Washington University School of Medicine, St. Louis, MO, USA; 3 Department of Molecular and Cellular Neurobiology, Center for Neurogenomics and Cognitive Research, Amsterdam Neuroscience, Vrije Universiteit Amsterdam, Amsterdam, the Netherlands; 4 Department of Psychiatry and Biobehavioral Sciences, David Geffen School of Medicine, University of California, Los Angeles, Los Angeles, CA, USA; 5 Intellectual and Developmental Disabilities Research Center, Washington University School of Medicine, St. Louis, MO, USA; 6 Present address: Department of Biological Sciences, University of Texas at Dallas, Richardson, TX, USA; 7 These authors contributed equally; 8 Lead contact

## Abstract

Within eukaryotic cells, translation is regulated independent of transcription, enabling nuanced, localized, and rapid responses to stimuli. Neurons respond transcriptionally and translationally to synaptic activity. Although transcriptional responses are documented in astrocytes, here we test whether astrocytes have programmed translational responses. We show that seizure activity rapidly changes the transcripts on astrocyte ribosomes, some predicted to be downstream of BDNF signaling. In acute slices, we quantify the extent to which cues of neuronal activity activate translation in astrocytes and show that this translational response requires the presence of neurons, indicating that the response is non-cell autonomous. We also show that this induction of new translation extends into the periphery of astrocytes. Finally, synaptic proteomics show that new translation is required for changes that occur in perisynaptic astrocyte protein composition after fear conditioning. Regulation of translation in astrocytes by neuronal activity suggests an additional mechanism by which astrocytes may dynamically modulate nervous system functioning.

## In brief

Across biological systems, localized and signal-mediated translation of proteins enables independent reactions by distinct compartments of the cell. It has been demonstrated previously that astrocytes have localized translation. Sapkota et al. demonstrate that astrocytic translation is regulated by neuronal activity, providing a mechanism for astrocytes to respond dynamically to nearby demands.

## Graphical Abstract

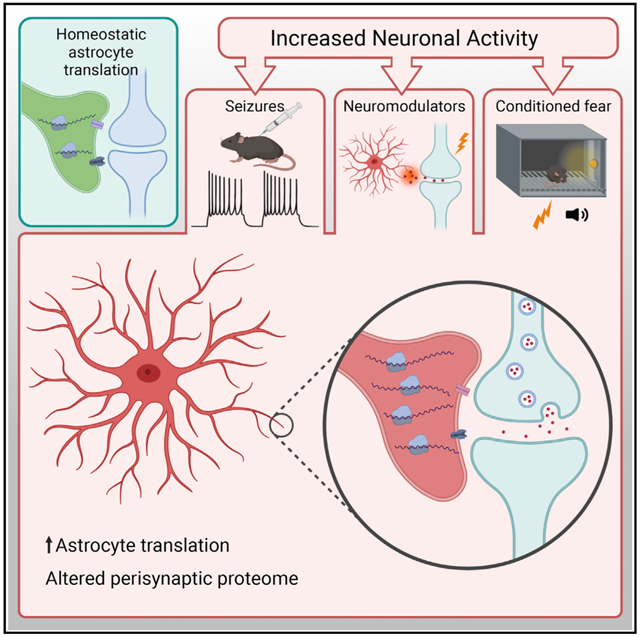

## INTRODUCTION

Activity-dependent transcription of genes and translation of mRNA are essential for long-term alterations of synaptic strength and memory consolidation. This essentiality is supported by classic studies using pharmacological inhibitors in cultures, acute slices, and behaving animals (Agranoff et al., 1966; Flexner et al., 1963; Kang and Schuman, 1996). At the transcriptional level, physiological activity, including Ca^2+^ influx, is known to activate transcription factors (TFs), such as cyclic AMP response element binding protein (CREB), resulting in a cascade of gene expression changes in neurons and glia (Hardingham et al., 2001; Pardo et al., 2017; Yap and Greenberg, 2018). In neurons, this involves induction of immediate-early genes such as *Fos* and *Jun*, TFs that support sustained alterations in synapses by increasing production of transcripts encoding synaptic proteins.

Traditionally, when profiling mixed cultures or brain after stimulation, most of the transcriptional response to synaptic activity has been presumed to be neuronal. However, it is well known that other cell types—astrocytes in particular—also respond to neuronal activity. Transcriptionally, neuronal maturation directly affects expression of the astrocyte glutamate transporter, GLT-1 (Perego et al., 2000; Swanson et al., 1997), and other genes (Hasel et al., 2017). Physiologically, astrocytes in the barrel cortex respond with increased cytosolic Ca^2+^ after stimulation of whiskers (Wang et al., 2006). Astrocytes increase *Fos* expression in a region-dependent manner (Chai et al., 2017). In a hippocampal long-term potentiation (LTP) RNA sequencing (RNA-seq) experiment, robust changes in transcripts associated with glia are also seen (Chen et al., 2017). There is also a clear induction of astrocyte gene expression when stimulated by neuronal activity in co-culture experiments (Goudriaan et al., 2014; Hasel et al., 2017), and single-cell RNA-seq experiments in the visual cortex demonstrate that astrocytes respond transcriptionally to visual stimulation as robustly as many classes of neurons (Hrvatin et al., 2018).

Although a transcriptional response to activity in mixed cultures and brain has been well studied, we (Dalal et al., 2017) and others (Chen et al., 2017; Cho et al., 2015) have shown that a distinct translational response also occurs. By sequencing mRNA specifically bound to ribosomes, we assessed the change in occupancy of mRNAs by ribosomes in response to sustained depolarization (Dalal et al., 2017). Although increased transcription of a gene often drove a corresponding increase in translation, at least 40% of the variance in translational response was independent of transcript levels. Likewise, Cho et al. (2015) applied ribosome footprinting to the hippocampi of animals exposed to a fear conditioning paradigm and quantified a substantial translational response (Cho et al., 2015). However, these studies were conducted on mixed populations of cell types, making the relative contributions of individual cell types difficult to assess. To this end, Chen et al. (2017) used translating ribosome affinity purification (TRAP) (Heiman et al., 2008; Sanz et al., 2009) to specifically enrich for ribosomes from hippocampal pyramidal neurons after LTP and sequenced the bound mRNA (TRAP-seq). The authors identified a time-dependent enrichment of a variety of transcripts on neuronal ribosomes, much of which was undetectable in parallel RNA-seq experiments from the same slices (Chen et al., 2017). This raises the question of whether astrocytes also undergo dynamic translational responses that were not apparent in prior transcriptional studies.

Likewise, whether astrocytes exhibit a programmed translational response to cues of neuronal activity has yet to be fully answered. A rapid translational response takes advantage of mRNA already present in the cell to produce new proteins without the time-consuming process of transcription. Because multiple ribosomes bind a single mRNA, translational regulation can rapidly amplify protein abundance. Finally, regulation of translation allows localized production of protein in specific subcellular compartments, such as near synapses, as observed in neurons (Cajigas et al., 2012; Hafner et al., 2019; Ouwenga et al., 2017, 2018; Steward et al., 2015), and in peripheral processes of astroglia, including the perisynaptic astrocyte process (PAP) (Boulay et al., 2017; Pilaz et al., 2016; Sakers et al., 2017).

We have shown previously that contextual fear memory learning in mice is accompanied by dynamic changes in the neuronal and astrocytic protein components of the hippocampal tripartite synapse (Rao-Ruiz et al., 2015). Astrocyte proteomic changes include downregulation of neurotransmitter transporters, such as SLC1A2/GLT-1, SLC1A3/GLAST, and SLC6A11/GAT3. Because new translation has been shown to be required for hippocampal memory (Flexner et al., 1963), these proteomic changes could be secondary to new translation. To what extent translation is required for activity-induced changes in PAP protein levels remains to be determined. In this work, we assess whether astrocytes also respond translationally to cues of neuronal activity *in vitro* and *in vivo*. We first use TRAP to test the hypothesis that a stimulus known to robustly activate neurons *in vivo*—a pentylenetetrazol (PTZ) induced seizure—will alter astrocyte translation. We found that ribosomal occupancy of hundreds of astrocyte transcripts is altered within minutes of seizure induction, whereas the majority of the corresponding genes had not yet changed transcriptionally. To better understand this result, we turned to imaging-based methods in acute slices to show alteration of translation in astrocytes in response to pharmacological manipulations that mimic aspects of neuronal activity. When performing these pharmacological studies in primary immunopanned astrocytes, the bulk of these responses is lost in the absence of neurons. Finally, proteomics analysis of synaptic fractions in contextual fear-conditioned mice was performed to determine the *in vivo* effect of blocked translation on the perisynaptic astrocyte proteome. We found that many of the proteomic changes induced by fear conditioning were blocked when translation was inhibited. These studies provide evidence of dynamic regulation of astrocyte translation by neuronal activity.

## RESULTS

### Acute seizure activity alters *in vivo* ribosome occupancy of transcripts in astrocytes

A key astrocyte function in the CNS is sensing and responding to neuronal activity (Charles et al., 1991; Perea et al., 2009; Schipke and Kettenmann, 2004). Astrocytes respond transcriptionally, morphologically, and functionally to the presence of neurons and neuronal activity (Goudriaan et al., 2014; Hasel et al., 2017; Hrvatin et al., 2018), but whether independent changes in their translatome also occur remains unexplored. Therefore, we tested whether a robust induction of neural activity would acutely alter the profile of mRNAs bound to astrocytic ribosomes *in vivo*.

**Figure 1.**
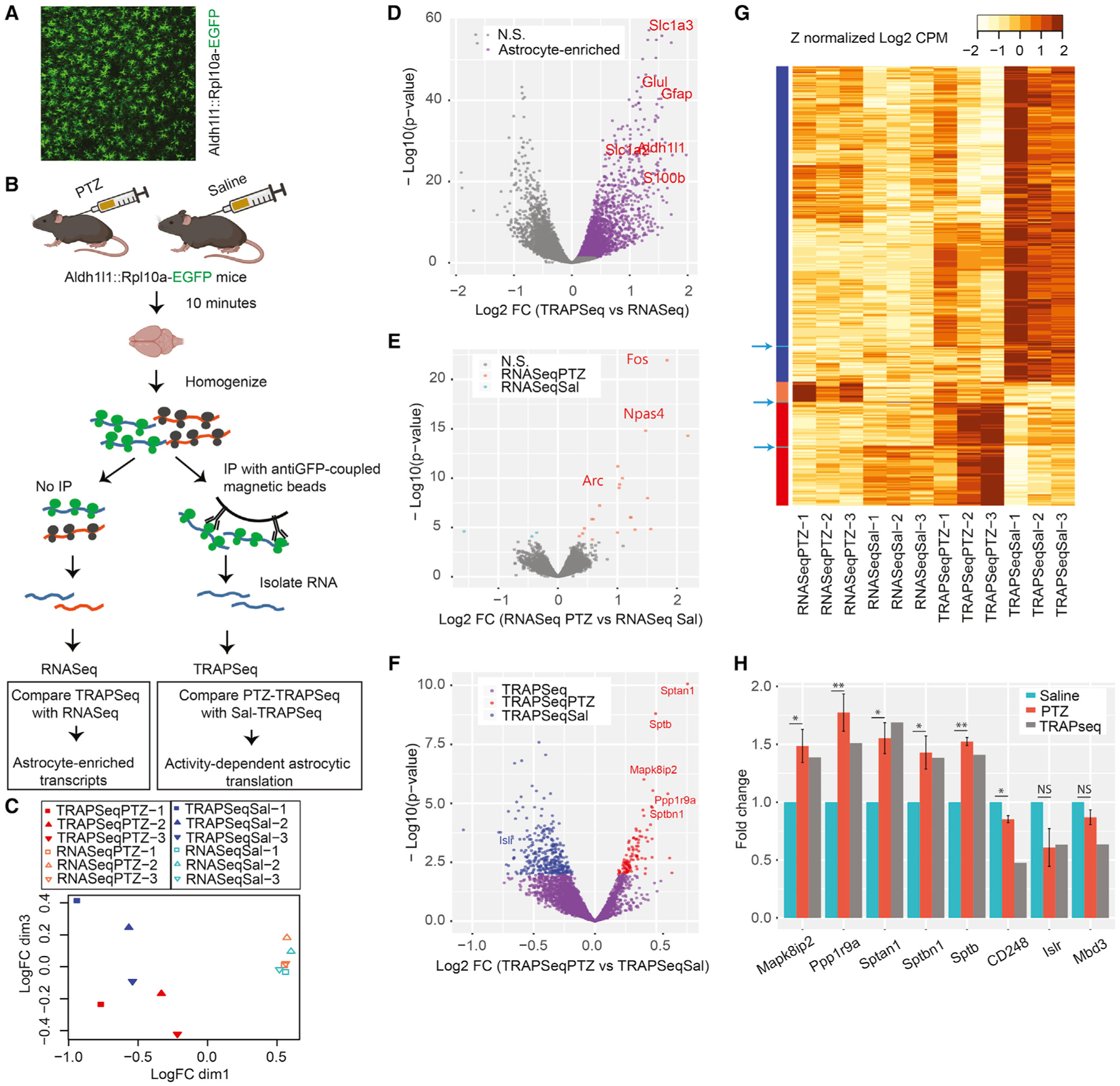
Seizure rapidly alters the translational profile of astrocytes (A) Representative image of an Astrocyte-TRAP mouse showing expression of the GFP-tagged ribosome construct in astrocytes across the cortex. (B) Schematic of immunoprecipitating astrocyte-specific mRNA with and without seizure induction. (C) Multidimensional scaling of 12 samples shows that astrocyte TRAP-seq samples are clearly separated from RNA-seq samples and that stimulated and unstimulated TRAP-seq samples are also distinguished in this unsupervised clustering approach. n = 3 mice per treatment. (D) Volcano plot comparing all TRAP samples with all RNA-seq samples defines transcripts enriched on astrocyte ribosomes (purple), including known astrocyte markers (red genes), as expected. (E) Volcano plot comparing RNA-seq from stimulated and unstimulated mice identifies the subset of transcripts responding rapidly to seizure induction (orange and blue transcripts, FDR < 0.1). Some immediate-early genes are marked. (F) Volcano plot comparing TRAP-seq from stimulated and unstimulated mice identifies the subset of TRAP enriched transcripts (purple) that were upregulated (red) or downregulated (blue) by seizure (FDR < 0.1). Genes of interest are labeled in the respective colors. (G) Heatmap of Z-normalized data for all transcriptionally upregulated (orange bar, left) or downregulated (cyan bars/arrows) transcripts and astrocyte translationally downregulated (blue bars) or upregulated (red bars) transcripts across all conditions. (H) PTZ- and saline-treated mice were subjected to qPCR, and fold changes were calculated as 2^−DeltaDeltaCt^. A mixed linear model followed by likelihood ratio test was used to test DeltaCt as a function of PTZ treatment. Corresponding TRAP-seq results are included for comparison. n = 3 mice per treatment. *p ≤ 0.05; **p ≤ 0.01.

We induced seizures in Astrocyte-TRAP mice, which express GFP-tagged ribosomes in all astrocytes (Figure 1A; Doyle et al., 2008), using PTZ, and harvested brains 8–10 min after PTZ or saline injection (Figure 1B). We focused on a short time interval to isolate the translational response independent of any PTZ-induced transcription. We collected transcripts bound to astrocyte ribosomes (TRAP-seq) and parallel whole-brain RNA (RNA-seq) from each sample and sequenced all samples to a depth of 28–43 million reads. Multidimensional scaling analysis shows that samples strongly cluster on one axis by whether they are from TRAP or RNA-seq and another axis by whether the TRAP samples come from PTZ- or saline-treated mice, illustrating reproducibility and indicating that a robust response is occurring on astrocyte ribosomes (Figure 1C). We next compared TRAP-seq samples with RNA-seq samples and confirmed that TRAP defined astrocyte-translated transcripts (Figures 1D and S1) and that these were significantly enriched for transcripts enriched by prior TRAP studies (Dougherty et al., 2012), immunopanning (Zhang et al., 2014), and nuclear sorting-based assessments of *in vivo* astrocyte gene expression (Reddy et al., 2017) (all p < 10E^−16^ by Fisher’s exact test). Thus, we confidently measured ribosome-bound mRNAs from astrocytes in PTZ- and saline-injected mice.

Next, we leveraged these data to determine whether these transcripts alter ribosome occupancy in response to stimulation. First, we directly compared the PTZ and saline RNA-seq data to test our assumption that using a short interval (8–10 min) would allow us to exclude most of the slower transcriptional response. The assumption largely held, but with few genes (23, at false discovery rate [FDR] < 0.1), showing a significant transcriptional response in this time window (Figure 1E; Table S2); these were largely immediate-early genes (e.g., *Npas4*, Log2FC = 2.2, FDR < 2E^−11^; *Fos*, Log2FC = 1.8, FDR < 1.3E^−18^; *Arc*, Log2FC = 1.02, FDR < 1.6E^−6^) known from the neuronal transcriptional response to synaptic activity. We contrasted this with the number of transcripts with altered ribosome occupancy by comparing PTZ TRAP with saline TRAP (Figure 1F). Because TRAP is an enrichment, not a perfect purification of astrocyte RNA, we conservatively filtered these to pursue transcripts that were also enriched by TRAP-seq over RNA-seq and removed the 23 that showed a transcriptional response, resulting in 417 transcripts (FDR < 0.1; Table S3; see Figure 1G and Table S4 for all transcripts and comparisons). Most of these showed minimal changes in ribosome occupancy because we limited PTZ action to 8–10 min to focus on acute responses. For instance, only four transcripts exhibited a fold change of ~1.5. Yet, using qPCR, we independently confirmed these changes for a majority of the candidates tested (Figure 1H). We confirmed 5 of 5 upregulated (*Mapk8ip2*, *Sptan1*, *Sptbn1*, *Sptb*, and *Ppp1r9a*) and 1 of 3 downregulated (*CD248* but not *Islr* and *Mdb3*, although the latter two still showed the expected trend) candidates and found PTZ treatment to be the factor driving these ribosomal changes (p < 0.05). Overall, we discovered that astrocytes do have a robust ribosomal response to stimulation *in vivo*, with at least 417 of the 3,410 astrocyte-enriched transcripts altering their ribosome occupancy and a larger proportion showing decreased (n = 315) rather than increased (n = 102) occupancy (Figure 1E). Thus, robust stimulation of neuronal activity induces immediate changes in astrocyte ribosome occupancy.

**Figure 2.**
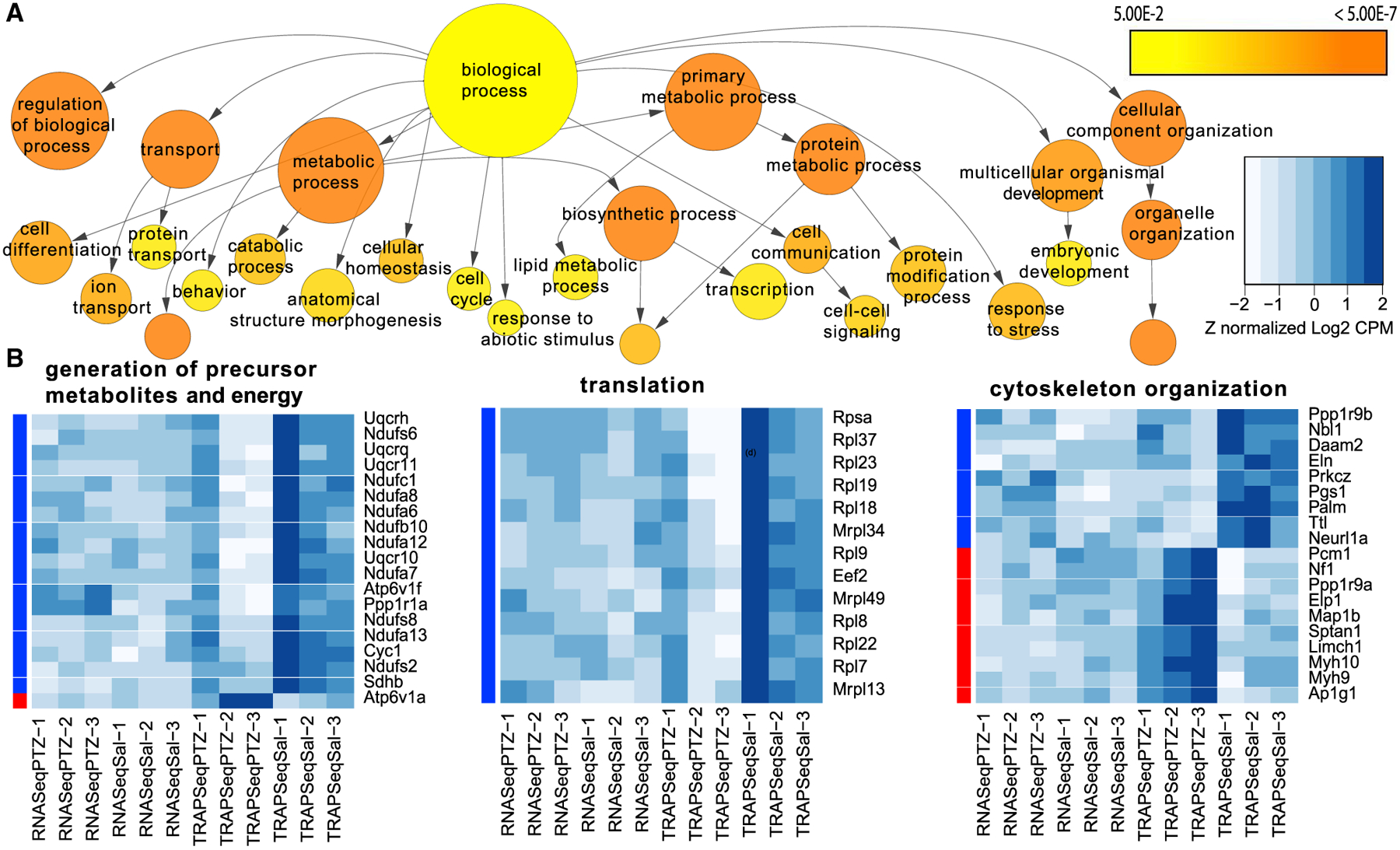
BiNGO analysis reveals biological processes significantly regulated in astrocytes after PTZ treatment (A) Exploratory GO pathway analysis of all regulated genes identifies trends for transcripts in key metabolic processes (e.g., mitochondrial, cytoskeletal, and ribosomal transcripts) with changes in ribosome occupancy. The color scale indicates significance for hypergeometric test. Category size is scaled to the number of genes. Arrows represent parent-child relationships in GO terms. (B) Heatmaps of the expression of the genes that make up three example categories show that most genes in the mitochondrial and ribosomal categories are downregulated with seizure. For cytoskeletal elements, there is a change between the genes in the category.

We then applied pathway analyses to these data to infer potential functions for the astrocytic translational response after seizure. First, examining all genes that were changed in either direction revealed an enrichment of processes such as cytoskeletal organization, translation, and energy generation (Figure 2A). Examination of individual genes driving these categories revealed that PTZ downregulated ribosomal occupancy of transcripts involved in energy generation and translation rather uniformly, whereas for transcripts involved in cytoskeletal organization, PTZ stimulated positive and negative changes in ribosomal occupancy on distinct sets of transcripts (Figure 2B). To understand the direction of effects on various categories, we repeated the analysis separately for up- and downregulated genes. Examining the downregulated genes, the gestalt view suggests a decrease in ribosomal occupancy of transcripts involved in a variety of metabolic pathways (Figure S1A). For example, ribosomal transcripts are altered (p < 3.03E^−7^, hypergeometric test, Benjamini-Hochberg corrected), as are key transcripts utilized by mitochondria (p < 8.37E^−14^), including members of the electron transport chain. This suggests that robust seizure activity in neurons triggers an immediate shift of protein production in astrocytes for metabolic functions. We next examined the upregulated genes and found robust increases in ribosome occupancy for transcripts encoding the cytoskeleton (p < 1.16E^−10^), notably actin binding proteins (p < 1.27E^−8^), a variety of motor proteins (p < 9.68E^−6)^, and ion transporting proteins (p < 1.19E^−5^) (Figure S1B; Table S5). The translational upregulation of cytoskeleton family transcripts suggests that an increase in neuronal activity could poise the astrocyte for increased perisynaptic process motility. This supports a previous finding demonstrating that an increase in intracellular Ca^2+^ induces astrocyte process motility (Molotkov et al., 2013) and that LTP induces PAP motility and retraction from the synapse (Henneberger et al., 2020; Perez-Alvarez et al., 2014). However, it is possible that these transcripts may reflect larger-scale motility or a combination of perisynaptic and non-perisynaptic cytoskeletal changes. Finally, the overrepresentation of transcripts in ion transport suggests a rapid homeostatic response to excessive neurotransmitter release induced by the seizure.

Transcript specific regulation of translation is often mediated by the interaction of RNA-binding proteins (RBPs) with specific sequences or RNA secondary structures, often found in the 5′ and 3′ untranslated regions (UTRs). We therefore tested the hypothesis that the regulated genes share sequence motifs. First, we examined broad features of the 5′ and 3′ UTRs and found that the upregulated transcripts generally had UTRs with a longer length and higher GC content (implying more stable secondary structures) than the downregulated transcripts (Figures S2A–S2D). Emboldened to consider the UTRs as being responsible for some of the transcript specific regulation, we tested the hypothesis that there are specific motifs shared across transcripts. We utilized the Analysis of Motif Enrichment (AME) tool within the Multiple Em for Motif Elicitation (MEME) suite (McLeay and Bailey, 2010) to screen for known vertebrate RBP target motifs in the upregulated and downregulated transcripts and compared their abundance. At FDR < 0.1, we found dozens of potential regulatory motifs differing between the sets, which could be grouped into families based on their sequence similarity (Figures S2E and S2F). This suggests that the regulation of translation of these transcripts is mediated by sequences found in UTR elements.

Finally, we sought to identify signaling pathways that may be upstream of these translationally regulated genes. We leveraged the COmprehensive Multi-omics Platform for Biological InterpretatiOn (COMPBIO) tool for contextual language processing to mine PubMed with the lists of translationally regulated genes and identify and cluster themes related to their gene products (https://gtac-compbio.wustl.edu/). COMPBIO reproduced aspects of the Gene Ontology (GO) analysis, including themes related to ribosomes, cytoskeletal components, and mitochondria. Beyond what was found by GO, it highlighted a surprising connection to neurodegenerative diseases driven by the Huntington’s disease-related genes *Htt* and *Smcr8* as well as some forms of intellectual disability driven by the neurofibromatosis genes *Nf1* and *Nf2* and the Cornelia de Lange syndrome genes *Smc3* and *Nipbl*. The upregulated transcripts were significantly enriched in recently empirically identified *de novo* autism genes (Fisher’s exact test, p < 0.0003) (Satterstrom et al., 2020) and a curated list of syndromic autism spectrum disorder (ASD)/intellectual disability (ID) genes (p < 0.05) (Abrahams et al., 2013). Finally, this analysis also highlighted Ras and mitogen-activated protein kinase (MAPK) signaling (Figure S3). These well-studied intracellular signaling pathways are known to be downstream of neuronal activity-induced neurotrophic factors, such as brain-derived neurotrophic factor (BDNF). BDNF is a known regulator of translation and synaptic plasticity in neurons (Leal et al., 2014), and astrocytes are also known to express BDNF receptors and respond to BDNF (Aroeira et al., 2015; Colombo et al., 2012; Wang et al., 1998). For example, astrocytes have been reported previously to increase nitric oxide production in response to BDNF (Colombo et al., 2012); we see increased ribosome occupancy of the key synthetase gene (*Nos1*) after stimulation (Table S3). Others have detected an increase in global translation in astrocytes after BDNF stimulation in co-culture with neurons (Müller et al., 2015). We therefore turned to an imaging-based analysis of translation to more directly assay the translational response in astrocytes to stimulation by BDNF and other molecular signals.

### Astrocytes respond translationally to cues of neuronal activity in acute slices

**Figure 3.**
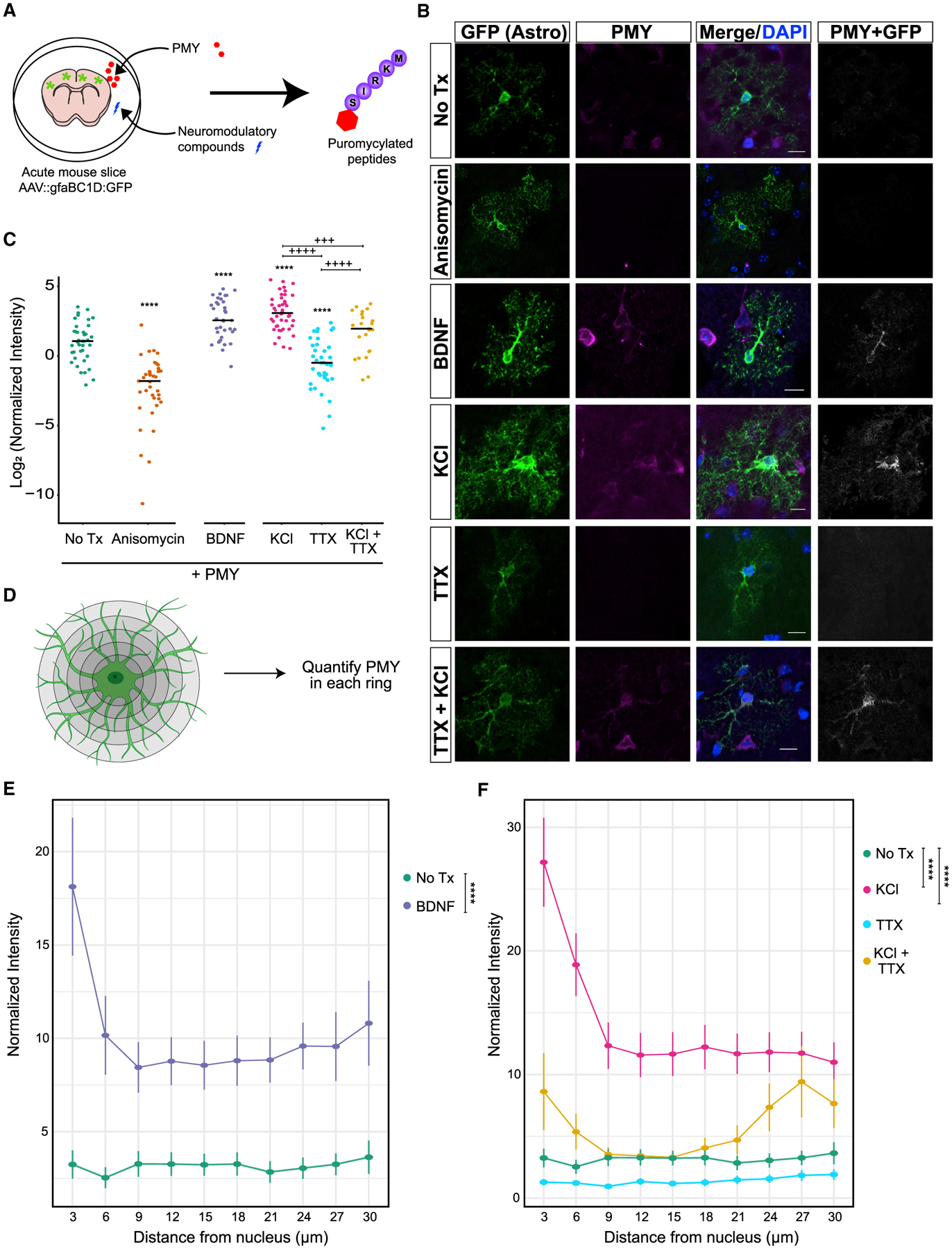
Astrocyte translation is modulated by BDNF and K^+^ (A) Schematic showing the *ex vivo* translation assay. Acute brain slices were treated with test compounds. The resulting translational changes were quantified using puromycin (PMY), which tags nascent peptides and serves as an epitope for the subsequent immunofluorescence. (B) Representative confocal images of post-natal day 21 (P21) cortical astrocytes after puromycylation. Astrocytes were labeled with AAV9:GFP (STAR methods) and incubated for 10 min with PMY and the indicated pharmacological manipulations. Immunostaining for GFP (green) and PMY (magenta) was performed. The PMY + GFP channel indicates colocalized pixels of PMY and GFP and was enhanced for publication. Scale bars, 10 μm. (C) Quantification of PMY intensity in GFP astrocytes. Normalized intensity was calculated by dividing PMY intensity by GFP area (pixels). ANOVA was performed to determine effect of condition; F(5,211) = 53.389, p < 2.2E^−16^. Post hoc pairwise t tests were performed. Asterisks indicate comparison with no Treatment (Tx) (PMY only), and plus signs indicate comparisons within KCl and TTX conditions. ^+++^p < 0.005, ****/++++p < 0.001. N_mice_ = 2–3 per condition, N_cells_ (condition) = 40 (no Tx), 37 (anisomycin), 36 (BDNF), 44 (KCl), 37 (TTX), and 23 (KCl + TTX). (D) Schematic of astrocyte PMY quantification as a function of distance. Concentric rings were drawn from the center of the nucleus starting at a radial distance of 3 μm and increasing to 30 μm maximum at an interval of 3 μm. The total PMY intensity was quantified within each ring and then normalized to the astrocyte area (GFP area) to account for differences in astrocyte volume in each ring. (E and F) Quantification of PMY intensity at increasing distance from the cell soma from the data in (I). Normalized intensity was calculated by dividing PMY intensity by GFP area (pixels). A linear mixed model was performed to account for random variation from each cell and each distance measured. Post hoc Tukey’s test is represented with asterisks. ****p < 0.0001. The cells used in this quantification are the same as in (C).

Although our TRAP experiment demonstrates that a seizure-inducing stimulus can alter ribosome occupancy of transcripts, it is not a direct measurement of translational response. PTZ-stimulated neurons produce a variety of cues that could induce translation, such as excess glutamate and extracellular K^+^ changes, and it is unclear to which of these the astrocytes are responding. Therefore, we assessed how neuronal activity acutely alters astrocyte translation by exposing acute brain slices to pharmacological manipulations that trigger or mimic aspects of neuronal activity and measured global translation in sparsely labeled astrocytes (Figure 3A). We first investigated whether BDNF stimulated translation of astrocyte transcripts in acute slices using immunofluorescence quantification of short-pulse puromycin (PMY) incorporation. PMY incorporates into the extending peptide chain on the translating ribosome and tags ongoing translation with an epitope that can be detected with immunofluorescence. BDNF induced a similarly robust increase in puromycylation in astrocytes (Figures 3B and 3C), and we confirmed that this was similar in magnitude to what was induced by PTZ (Figure S4A). These changes were consistent with increased translation because preincubation with another inhibitor of translation, anisomycin, blocked the PMY signal. BDNF transcription and secretion are regulated by neuronal activity (Balkowiec and Katz, 2000; Dieni et al., 2012; Zafra et al., 1990); thus, we hypothesized that globally altering neuronal firing regulates astrocyte translation. Therefore, we depolarized the slices by elevating K^+^ using 5 mM KCl, a manipulation known to depolarize neurons and increase action potential frequency (Paluzzi et al., 2007; Rienecker et al., 2020). KCl alone also robustly increased PMY incorporation in astrocytes, indicating that increased neuronal firing stimulates nascent protein synthesis in astrocytes (Figure 3B). Consistent with previous reports (Song and Gunnarson, 2012), we noted an increase in cell size after treatment with KCl (Figure S4B), suggesting that water permeability is also increased in astrocytes. However, even after normalizing the PMY intensity to cell size, quantified by astrocyte-GFP area, we still detected a robust increase in translation (Figure 3C), which argues against the possibility that increased cell size explains the greater PMY intensity. We next asked the reciprocal question of whether silencing neuronal firing results in a decrease in astrocyte translation by blocking neuronal spiking using tetrodotoxin (TTX). TTX alone significantly decreased PMY intensity in astrocytes (Figure 3C), suggesting that at least some of the basal level of translation in astrocytes is dependent on spontaneous activity of surrounding neurons. However, activity-independent translation also occurs in astrocytes because TTX-treated slices contained significantly more PMY compared with anisomycin-treated slices (Figure 3C). Because KCl also depolarizes astrocyte membranes, we next investigated whether induction of astrocyte translation by KCl was dependent on neuronal firing and subsequent neurotransmitter release. Therefore, we blocked neuronal firing with TTX prior to KCl stimulation. Pairing KCl treatment with TTX significantly inhibited most of the KCl-induced increase in translation. However, translation was still higher than when TTX was used alone (Figure 3C). Thus, these findings suggest that K^+^-induced translation in astrocytes has components that are dependent and independent of neuronal activity.

### Activity-dependent astrocyte translation is non-cell autonomous

**Figure 4.**
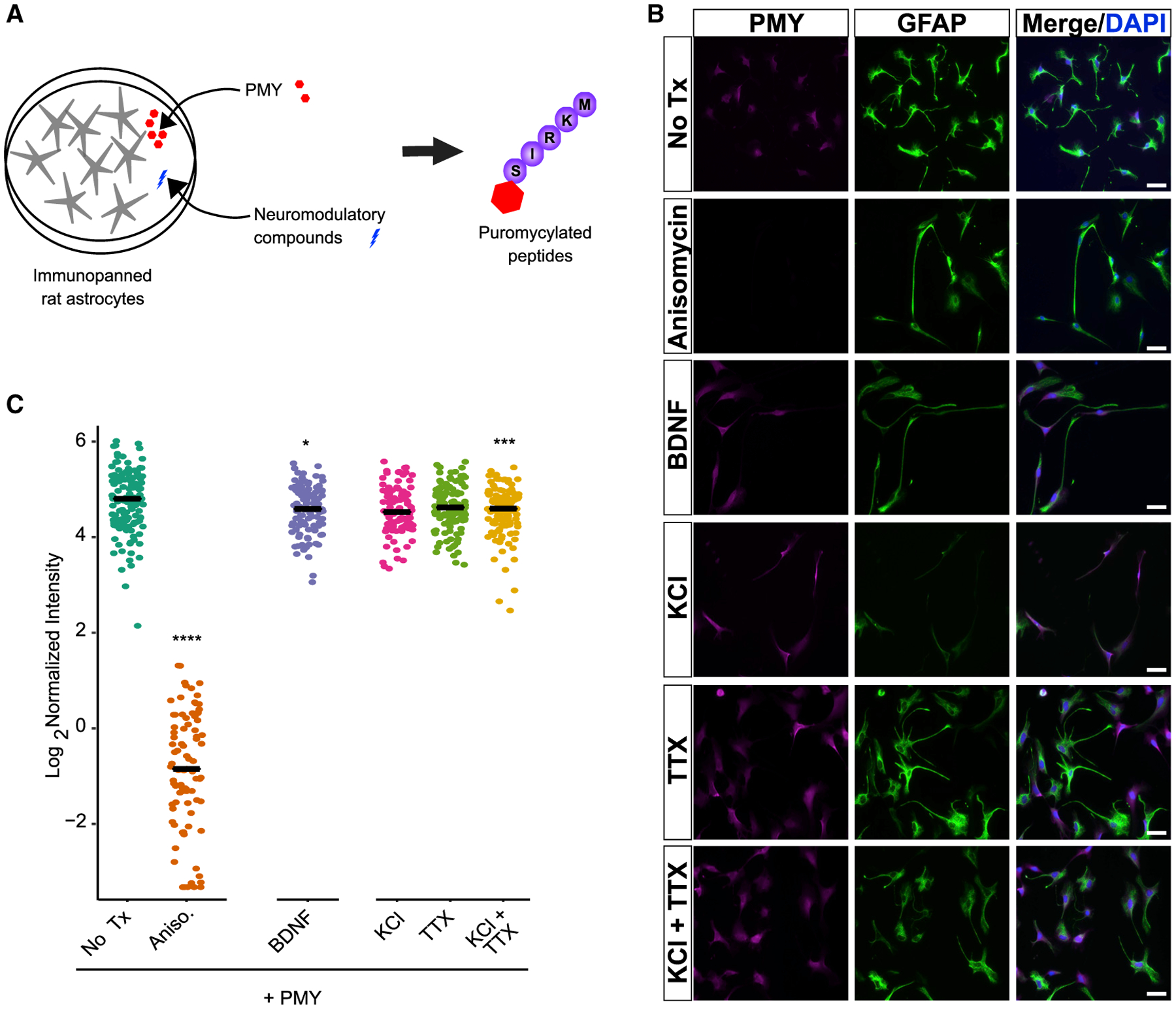
The effect of BDNF and KCl on astrocyte translation requires neurons (A) Schematic of the assay for astrocyte translation. Purified astrocytes are treated with test compounds, and the resulting translational response is measured using PMY, which tags nascent peptides and is visualized for the subsequent immunofluorescence. (B) Representative fields of immunopanned astrocytes, stained for PMY and GFAP. Scale bar, 50 μm. (C) Quantification of PMY intensity in astrocytes. Mean intensity (signal/area) was calculated for individual cells. Pairwise t tests were performed compared with no Tx. ****p < 0.001, ***p < 0.005, *p < 0.05. N_cells_ (condition) = 129 (no Tx; i.e., PMY only), 87 (anisomycin), 97 (KCl), 120 (TTX), 104 (BDNF), 111 (KCl + TTX).

The TTX experiments provide evidence that the presence of neurons and neuronal action potential firing is largely required for the KCl response in astrocyte translation. However, it does not rule out the possibility that other signaling pathways from neurons, independent of spiking, drive some aspect of the astrocyte response. We therefore examined this possibility by separating astrocytes from neurons and assessing their translational response to the same panel of chemical modulators (Figure 4A). We utilized a primary culture system in which astrocytes are immunopanned from dissociated rat cortices (Foo et al., 2011; Zhang et al., 2016). In this system, astrocytes exhibit morphological, physiological, and transcriptional profiles similar to those *in vivo* and, thus, enable a more direct comparison with our *ex vivo* data than traditionally cultured astrocytes. As before, we found that 1 mM anisomycin was sufficient to block the PMY signal in this system (Figures 4B and 4C). However, we found that no other manipulation resulted in a significant upregulation of the PMY signal *in vitro*. In contrast to our slice findings, we found a modest but significant downregulation of translation when astrocytes were treated with BDNF (Figure 4C). This finding is reinforced by previous work using comparable methods, supporting the idea that astrocyte translational regulation is mediated by neuronal firing and/or neuronal contact (Müller et al., 2015). We found another modest but significant decrease in translation from the combination of KCl and TTX but no effect of TTX or KCl alone (Figures 4B and 4C). We confirmed that, similarly, treatment with PTZ had no significant effect on translation (Figure S4C). Thus, although it is not the only explanation, these data are consistent with activity-dependent signaling from neurons inducing astrocyte translation.

### The astrocyte periphery also responds translationally to cues of neuronal activity

Astrocytes have been shown to have localized translation at their perisynaptic (Sakers et al., 2017) and vascular contacts (Boulay et al., 2017). We hypothesized that sensing chemical changes at the synapse stimulates localized translation in astrocytes in an activity-dependent manner. As a first pass, we used the images from Figures 3A–3C and quantified the abundance of PMY signal in concentric rings drawn at increasing radial distances from the cell soma. To account for the difference in astrocyte territory size within each ring, we normalized the PMY intensity levels to the GFP area (Figure 3D). Although BDNF and KCl strongly increased translation in the cell soma, there was a considerable increase in PMY signal to the distal processes (Figure 3E). Given the short incubation and subsequent washout of PMY, this distal labeling is consistent with localized translation. Although TTX reduced translation throughout the astrocyte, the combination of KCl and TTX substantially reduced translation in the soma and proximal processes but not in the distal branches (Figure 3F). These data support a model where nascent translation in subcellular regions of the astrocyte responds differently to cues of neuronal activity. Although we determined that there are subcellular differences, one caveat to this analysis is that we cannot say which pixels from the astrocyte are precisely perisynaptic at this resolution.

Overall, we show in our slice experiments that astrocytes respond to manipulated cues of neuronal activity with a translational response. However, these pharmacological manipulations do not show whether this translational response occurs with physiological manipulations, nor the resulting changes in protein levels. They also do not measure changes specifically to the synaptic fraction. Thus, we turned to a different system to address these concerns.

### Activity-dependent astrocyte translation alters the perisynaptic proteome under physiological conditions

Classic studies blocking new translation in the hippocampus during fear conditioning conclusively demonstrate that translation is required for memory consolidation (Debiec et al., 2002). We therefore set up an experiment to determine whether translation is important for alterations of the local perisynaptic astrocytic proteome we detected previously (Rao-Ruiz et al., 2015) after physiological stimulations that induce memory consolidation. We hypothesized that, if new translation is required for the perisynaptic proteomic alterations driven by fear conditioning, then blocking translation with cycloheximide (CHX)_would block the changes observed previously.

**Figure 5.**
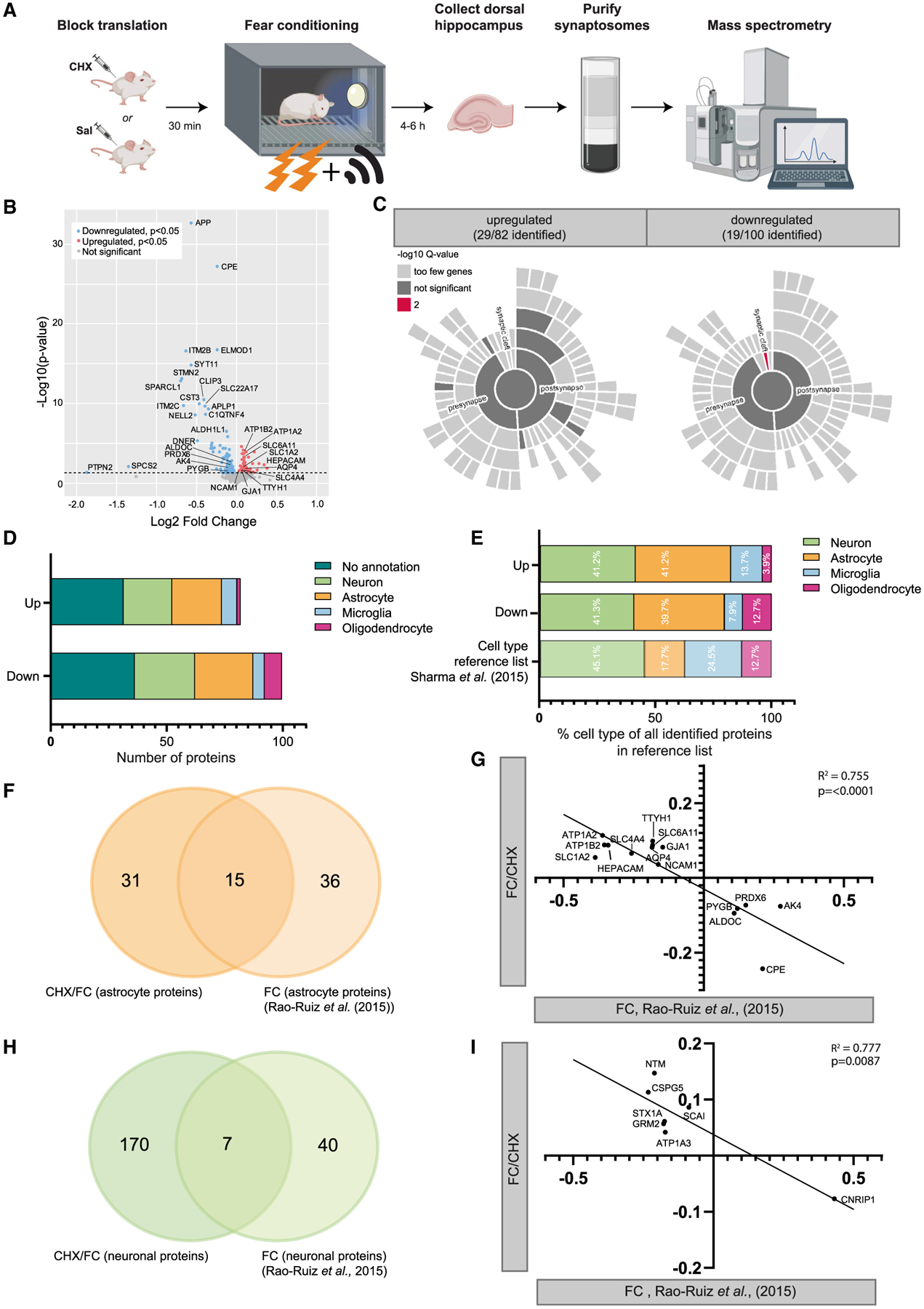
The astrocytic perisynaptic proteome is altered when translation is inhibited by CHX Tx (A) Experimental design of blocked translation by cycloheximide (CHX) before fear conditioning, followed by proteomics analysis on hippocampal synaptosomes. (B) Volcano plot depicting proteomics data with expression changes in Fear Conditioned CHX-treated mice (n = 9) (FC/CHX) compared with Fear Conditioned non-treated controls (n = 10) (FC/CTL) (x axis, log2) and statistical significance (y axis). Downregulated significant (p < 0.05) proteins are shown by individual red dots and upregulated proteins by blue dots. Non-significant observed proteins are shown in gray. Significance threshold is represented by the dashed line. (C) Sunburst plots showing the annotation in synaptic compartments using SynGO. Color coding is based on FDR-corrected p values (q values). (D) Cell enrichment analysis based on cell-type-specific proteomics data by Sharma et al. (2015). Data are provided for all upregulated and downregulated proteins separated. Each bar represents a different cell type. (E) Fractions of annotated cell types for proteins regulated by CHX Tx compared with fractions of cell types identified in the reference list for cell type enrichment analysis by Sharma et al. (2015). Percentages are based on all identified regulated proteins with a cell type annotation, divided by “upregulated” and “downregulated.” (F) A Venn diagram showing similarities between astrocyte proteins regulated by FC/CHX and proteins regulated by FC only (Rao-Ruiz et al., 2015). (G) Correlation plot showing high inverse correlation for changes in astrocyte protein levels caused by CHX (FC/CHX – FC/CTL, x axis) versus FC (Rao-Ruiz et al., 2015; y axis). (H) Venn diagram showing similarities between neuronal proteins regulated by FC/CHX and proteins regulated by FC only (Rao-Ruiz et al., 2015). (I) Correlation plot showing high inverse correlation for changes in neuronal protein levels caused by CHX (FC/CHX – FC/CTL, x axis) versus FC only (Rao-Ruiz et al., 2015; y axis).

We first replicated that CHX before fear conditioning blocks hippocampus-dependent memory formation in mice (Figure S5). We then treated mice with CHX or saline, fear conditioned both groups, and conducted unbiased proteomics on synaptic fractions from the dorsal hippocampus collected 4 h later as done by Rao-Ruiz et al. (2015) (Figure 5A). Across all samples, more than 3,300 proteins and more than 19,000 peptides were detected, with more than 15,000 of these being identified in all samples (Methods S1). The data were reproducible across all replicates, as shown by the analysis of abundance distributions, retention times, and coefficient of variation computations (Methods S1). With this, we concluded that the data were of high quality and reliable to use for analysis.

A total of 182 proteins were significantly differentially regulated (p < 0.05) in the CHX-treated group (FC/CHX) compared with saline controls (FC/CTL). One hundred of these proteins were found to be downregulated, and 82 to be upregulated (Figure 5B). A list of all detected proteins is present in Table S7. A GO analysis using BiNGO (Biological Networks Gene Ontology) was performed on the regulated proteins compared with astrocyte-expressed genes and identified coherent changes in proteins involved in ion regulation as well as glycogen and glutathione modulation (Figure S6). This GO categorical enrichment was driven by the astrocytes because the same analysis subsetting on the neuronal genes identified no significant categories.

We first determined the regulation of proteins that were of synaptic origin by using the Synaptic Gene Ontology database SynGO (Koopmans et al., 2019). A significant fraction of the changed proteins (48 of 182) were found in the SynGO database (Figure 5C), mostly split evenly between the presynapse and postsynapse compartment, because no significant enrichment was observed when comparing these two compartments. However, the category “synaptic cleft” was enriched in the downregulated proteins (p = 1.83E^−3^ after FDR correction) compared with other synaptic compartments. This involved the proteins APOE, SPARCL1, C1QL2, and C1QL3, which are secreted proteins and of which at least the first two have been shown to be highly expressed by astrocytes (Sharma et al., 2015). To explore the probable cellular origin of all 182 of the regulated proteins, we performed a cell-type-specific enrichment analysis based on high-resolution proteomics data on the single cell types of Sharma et al. (2015) (Figure 5D). A large proportion of the regulated proteins are enriched in neurons (p < 0.0001), as expected. However, an equally sized proportion of regulated proteins is enriched in astrocytes (Fisher’s exact test, p < 0.0001). The observed fraction of astrocyte enriched proteins was more than expected by chance, based on the percentage of astrocytic proteins present in the reference list (Figure 5E), unlike the minor fraction also found for microglia and oligodendrocyte enriched proteins. These findings are in line with previous observations that PAP proteins can be detected in synaptosomes (Carney et al., 2014; Chicurel et al., 1993; Rao-Ruiz et al., 2015; Shavit et al., 2011) and indicate that new translation affects the proteome of the neuronal as well as the astrocytic elements of the activated synapse.

To obtain further insight into the regulation of translated PAP proteins by synaptic activity during memory consolidation, we compared our data with the dataset published earlier by Rao-Ruiz et al. (2015). This study identified changes in the synaptic proteome, including 51 astrocyte enriched proteins, after contextual fear conditioning. Interestingly, 15 of the 46 CHX-regulated astrocyte proteins here were found in this prior study to be regulated by contextual fear conditioning alone (Figure 5F). Consistent with the hypothesis that new translation is required for the previously observed synaptic and perisynaptic changes after fear conditioning, a strong inverse correlation was found for the direction of regulation of astrocyte proteins between the two datasets (Pearson R^2^ = 0.755, p < 0.0001; Figure 5G). Specifically, most of the astrocyte proteins were found to be upregulated by CHX (FC/CHX versus FC/CTL) but downregulated by fear conditioning alone (dataset from Rao-Ruiz et al., 2015). A similar observation was made for the directional regulation of neuronal proteins, although a much smaller overlap was found between neuronal proteins regulated by CHX or fear conditioning alone (Figures 5H and 5I). For a large portion of the astrocyte proteins regulated by fear conditioning and FC/CHX, perisynaptic localization has been described previously: SLC1A2 (Chaudhry et al., 1995; Cholet et al., 2002; Minelli et al., 2001), SLC6A11 (Melone et al., 2015; Minelli et al., 1996), AQP4 (Nagelhus et al., 2004; Nielsen et al., 1997), ATP1A2 (Cholet et al., 2002), and PYGB (Richter et al., 1996).

These data indicate that CHX treatment before shock is affecting translation in astrocyte perisynaptic processes and that blocking translation blocks many of the normal changes that follow learning. This includes many proteins that could function in formation of memory upon physiological stimulation.

## DISCUSSION

Proper nervous system development and function require precise spatiotemporal translation of proteins. There has been a recent focus on identifying transcriptional programs correlated with important developmental milestones (Kalish et al., 2018) and neuronal activity (Hrvatin et al., 2018). Neuronal activity can induce such transcription in a cell-autonomous fashion, which has been shown to be important for synaptic plasticity and is classically mediated through activation of factors such as CREB (Yap and Greenberg, 2018). Although CREB also induces activity-dependent transcriptional changes in astrocytes (Hasel et al., 2017; Pardo et al., 2017), neuronal activity-regulated translation in astrocytes has been mostly overlooked. Here we show, in acute slices, that overall astrocyte translation changes after treatment with neuronal activity modulators only in the presence of neurons. We profile the changes in ribosome occupancy in astrocytes acutely after seizure induction by TRAP-seq and find a robust translational response, with hundreds of transcripts showing regulation. Pathway analyses highlight independent roles of upregulated and downregulated genes, and analyses of sequence features indicate that some of this regulation may be driven by shared motifs in UTR sequences. Literature mining tools suggest regulation by BDNF and some relationship with genes implicated in neurodegeneration and neurodevelopmental disorders. Several astrocyte genes involved in ASD appear to show acute regulation of translation in astrocytes in response to neuronal activity.

It is clear from this study that astrocytes have a robust and transcript-specific translational response to neuronal activity across assays and regions. We found that, similarly to PTZ, BDNF and KCl induce translation in astrocytes. In agreement with our findings, a study using fluorescent non-canonical amino acid tagging (FUNCAT) (Dieterich et al., 2010), an analogous method to puromycylation, found similar results of BDNF *in vitro* on astrocyte translation (Müller et al., 2015). Although the live slice experiments were not designed and powered to find region-specific translational responses (in part because few hippocampal astrocytes were transduced with our labeling approach), hippocampal cells followed the same trends as cortical cells (Figure S5), suggesting that astrocytes produce a translational response across regions. KCl stimulation—a manipulation that can trigger firing and model the high K^+^ concentrations that occur transiently after neuronal firing—has components dependent on and independent of neuronal action potentials, as shown by TTX blockade. This may mean that there are distinct transcripts regulated by each neuronal signal, perhaps secondary to distinct secondary messenger events. This is supported by the discovery of motifs in subsets of the transcripts. We found motifs for over a dozen different RBPs in each transcript list—more than could be readily pursued experimentally here. Because many of these motifs are quite similar across RBPs, it can be a challenge to identify which, if any, of the known RBPs may play a role in this context. It is possible that an RBP molecular code is defined by discrete signaling pathways that, upon activation, alter translation of specific mRNAs downstream of each RBP. On the other hand, there could be a less specific mechanism where a variety of RBPs have redundant roles and work in a larger ensemble. For example, the set of RBPs identified as enriched in the upregulated transcripts is remarkably similar to the motifs found in our recent study of Celf6 binding targets (Rieger et al., 2020), despite the actual transcripts being distinct. Celf proteins have a role in forming RNA granules for mRNA storage and/or decay, which can contain numerous RBPs and mRNAs interacting non-specifically through phase separation mediated by disordered domains. This suggests that transcripts enriched in binding for this selection of proteins may be more likely sequestered into a common granule awaiting release and translation after neuronal activation. Common to both studies, there was a GC bias in the lists, and thus there could be shared preference for GC-binding RBPs in both cases. More sophisticated microscopy to track individual mRNAs and a targeted proteomics study of some of these proteins in astrocytes may elucidate which RBPs play a role in the regulation described here. This could eventually help test whether distinct signals regulate specific transcripts or whether there is a more coordinated action across RBPs.

Comparing the slice PMY experiments with the astrocyte monoculture experiments suggests that most of this translational response requires the presence of neurons. This may reflect an acute response in astrocytes to signaling molecules released by spiking neurons or longer-term gene expression changes mediated by maturation in the presence of neurons. Prior work comparing astrocyte monoculture with co-culture experiments indicates that the BDNF receptor is upregulated moderately by long-term culture with neurons (Nrtrk2, 1.98-fold, p.adjust < 2.2E^−5^), whereas there is a dramatic rearrangement of K^+^ channel expression, with several increasing (Kcna2, Kcnk1, Kcnj16, Kcnn2, and Kcnn3) and several decreasing (Kcnj12, Kcns3, Kcnq4, Kcnd3, and Kcnc3; all p.adjust < 0.05) (Hasel et al., 2017). Regardless of whether the ability to respond is mediated by an acute or long-term presence, the slice experiments make it clear that neuron spiking is needed acutely for the most robust KCl response.

It is interesting to consider the relationship between global translation regulation and the potential for local translation regulation at peripheral astrocyte processes and endfeet (Boulay et al., 2017; Sakers et al., 2017). We detected robust activity-dependent translation in astrocyte distal segments after a brief pulse of PMY and subsequent washout and fixation to prevent sustained nascent peptide labeling. Addition of TTX to KCl-treated slices blunts the effect of KCl in the soma and proximal area but not in more distal regions. Without knowing the location of synapses relative to the astrocyte territory, we cannot claim that this localized translation is perisynaptic. However, our subsequent mass spectrometry-based proteomics of synaptic fractions after physiologically stimulating the dorsal hippocampus revealed a requirement for new translation for 46 proteins that are enriched in astrocytes, and many have prior evidence of local translation (Sakers et al., 2017). Although methods do not yet exist to inhibit just local translation without affecting the rest of the cell, these results strongly suggest that local translation in astrocytes plays a role in dynamically adapting the PAPs to the demands associated with learning at synapses. Likewise, it is intriguing that there is a highly significant overlap (p < 10E^−16^, Fisher’s exact test) between the transcripts identified as PTZ stimulated, particularly those that are downregulated, and those described previously as bound to astrocyte ribosomes in perisynaptic fractions (Sakers et al., 2017). This suggests that some of these transcripts may be stalled on ribosomes in the periphery, awaiting a signal secondary to neuronal activation, at which point they are rapidly translated and subsequently degraded, similar to what has been reported for Arc in neurons (Farris et al., 2014). This could provide a substrate for local and activity-dependent production of certain proteins in specific processes of a given astrocyte and would require a highly localized signal to activate such translational activity in a process-specific manner. Candidates could be Ca^2+^ transients in microdomains or regions of elevated kinase activity downstream of G protein-coupled receptors or receptor tyrosine kinase cell surface receptors, such as those activated by BDNF. Our findings here motivate future studies aimed at understanding the potential for subcellular localization of activity-dependent translation in astrocytes.

Re-examining our puromycylation studies reveals that some combinations of stimuli (e.g., KCl and TTX) could skew translation in one compartment compared with another (Figure 3F), consistent with a cue-specific regulation of local translation. Mass spectrometry-based proteomics of synaptic fractions after a fear conditioning stimulation paradigm of the dorsal hippocampus revealed a requirement for new translation for the levels of at least 182 proteins, a significant fraction of which are expressed in astrocytes and have prior evidence of activity-dependent changes in protein levels in the synaptic fraction (Figure 5; Rao-Ruiz et al., 2015). For instance, contextual fear memory-induced upregulation of the astrocyte proteins ALDOC, PYGB, PDX6, CPE, and AK4 has been found to be partially dependent on translation. Reduced expression of many of these proteins has been associated with impaired memory (Ji et al., 2017; Mathieu et al., 2016; Opii et al., 2008; Phasuk et al., 2021). The contextual fear memory-induced downregulation of numerous astrocyte proteins (Figure 5G; Rao-Ruiz et al., 2015) has been found to be blunted by a translation block. This included established PAP proteins involved in neurotransmitter uptake (SLC1A2 and SLC6A11) and ion balance (ATP1A2) that serve in balancing neuronal activity during and after the cellular- and network-level changes involved in learning. It remains to be determined how downregulation of these proteins is dependent on translation, but it may involve diverse mechanisms, such as protein degradation, reduced PAP localization (Mazaré et al., 2020; Murphy-Royal et al., 2015), reduced ribosomal occupancy (Mazaré et al., 2020), or activity-induced PAP retraction (Henneberger et al., 2020; Perez-Alvarez et al., 2014). Regardless of the mechanism, these studies provide collective evidence arguing that astrocyte translation can be regulated by neuronal activity to dynamically and rapidly alter the contribution of the astrocyte process to the perisynaptic milieu.

### Limitations of the study

The current study does have limitations that should be mentioned. First, TRAP is a method for enrichment, not perfect purification. Therefore, we conservatively limited our analysis for translationally regulated transcripts to those significantly enriched in astrocytes. It is possible that, with additional strategies for enrichment, we could identify more transcripts that are changing ribosome occupancy. However, even with the current criteria we were able to identify hundreds of transcripts. Although assessment of ribosome occupancy does indicate regulation of translation, it is not a measurement of actual nascent protein production. Still, when initially validating the efficacy of the TRAP method, Heiman et al. (2008) found that the change in translational efficiency of ferritin mRNA in response to iron treatment was comparable between samples processed via TRAP and traditional polysome centrifugation methods, suggesting that TRAP can be a reliable proxy for changes in translation. However, it is not yet known whether TRAP can discriminate between monosomes and polysomes in the brain; thus, the enrichment might reflect an increase in stalled ribosomes on a given transcript in stress granules rather than a recruitment of new ribosomes and increased translation. Therefore, one should be cautious about inferring direction of changes in protein levels from changes in TRAP alone. There was significant (Fisher’s exact test, odds ratio [OR] 2.3, p < 0.0054) but not overwhelming overlap with our later proteomics studies of new protein translation and perisynaptic translation. This is still a notable amount of overlap, given the second limitation that TRAP studies were conducted on the whole brain, whereas our proteomic studies were hippocampus specific (PTZ does induce neuronal activity in the hippocampus but also in the cortex and amygdala; Yang et al., 2019). On one hand, this indicates that the overall phenomena of activity-induced changes in astrocyte translation is robust across regions. For our slice studies, we attempted to evaluate astrocyte translation in hippocampal and cortical cells. On the other hand, astrocyte subtypes with distinct functional and transcriptional signatures have been characterized between the cortex and hippocampus (Batiuk et al., 2020). Thus, it will be interesting in the future to design studies where TRAP, puromycylation, and proteomics results in astrocytes after neuronal activity can be compared within and between regions. This will allow determination of whether translational responses to neuronal activity are fine tuned in distinct astrocyte subtypes. Finally, our PMY labeling in acute slices was analyzed with diffraction-limited technologies that cannot resolve individual PAPs and synapses. Future studies using methods such as stochastic optical reconstruction microscopy (STORM) or expansion microscopy, may provide the resolution needed to make more precise quantification of PAP versus synaptic translation. Despite these limitations, overall, the evidence strongly indicates that astrocytes have a dynamic translational response to neuronal activity.

## STAR★METHODS

### RESOURCE AVAILABILITY

#### Lead contact

Further information and requests for resources and reagents should be directed to lead contact, Joseph Dougherty (jdougherty@wustl.edu).

#### Materials availability

This study did not generate new unique reagents.

#### Data and code availability

Sequencing data have been deposited at GEO: GSE147830 and are publicly available as of the date of publication. Original code has been deposited at BitBucket: 10.5281/ZENODO.7054545, and original proteomics code has been deposited at GitHub: https://doi.org/10.5281/ZENODO.7054911 and is publicly available as of the date of publication. GEO accession numbers and code availability are listed in the key resources table. Any additional information required to reanalyze the data reported in this paper is available from the lead contact upon request.

### EXPERIMENTAL MODEL AND SUBJECT DETAILS

#### Mouse lines

All procedures were performed in accordance with the guidelines of Washington University’s Institutional Animal Care and Use Committee. Mice were maintained in standard housing conditions with food and water provided ad libitum and crossed at each generation to wildtype C57BL/6J mice from Jackson labs. The TRAP line was B6.FVB-Tg(Aldh1L1-EGFP/Rpl10a)^JD130Htz^, hereafter Astrocyte-TRAP (Doyle et al., 2008). Unless otherwise stated, mice of both sexes were used, with sex randomly assigned to each condition. Early postnatal tissue was harvested at P21 for all PTZ-TRAP studies. For live slice experiments, mice were injected with 2 μL of AAV9-CBA-IRES-GFP virus at postnatal day 1–2 (P1-P2) and acute cortical slices were prepared at P21. Fear conditioning studies were conducted on adult wildtype C57BL/6J mice (>P60).

### METHOD DETAILS

#### PTZ treatment and TRAP

Three-week-old Astrocyte-TRAP mice of both sexes were intraperitoneally injected with PTZ at 60 mg/kg (Sigma, 5 mg/mL stock solution in normal saline). Mice showing persistent convulsions at 8–10 min were subjected to rapid decapitation using decapicones (Braintree Scientific) for harvesting the brains. Control mice were injected with 110 μL normal saline (Sal) and decapitated similarly after 10 min. The harvested brains were snap-frozen in liquid nitrogen and stored at −80°C for TRAP. Six mice (three per treatment) were used.

TRAP was performed as described (Heiman et al., 2008) with a few modifications. Briefly, the brains were homogenized in ice in a buffer (20 mM HEPES pH 7.4, 150 mM KCl, 5 mM MgCl2, 0.5 mM dithiothreitol, 100 μg/mL cycloheximide (CHX), protease inhibitors, and RNase inhibitors). The lysates were cleared by centrifuging at 2000 × g for 10 min at 4°C and treated with DHPC (to 30 mM, Avanti) and NP-40 (to 1%, Ipgal-ca630, Sigma) for 5 min in ice. Lysates were further cleared by centrifuging at 20,000 × g for 15 min at 4°C. A 1/10^th^ volume of the cleared lysate was saved as the input control and used to generate RNAseq samples, and the rest was mixed with protein L-coated magnetic beads (Invitrogen), previously conjugated with a mix of two monoclonal anti-GFP antibodies (Doyle et al., 2008), and incubated with rotation for 4 h at 4°C. Beads were washed 5 times with a high-salt buffer (20 mM HEPES pH7.4, 350 mM KCl, 5 mM MgCl2, 1% NP-40, 0.5 mM dithiothreitol, and 100 μg/mL CHX) and finally resuspended in 200 ul normal-salt buffer (150 mM KCl, otherwise as above).

RNA was extracted from the input and TRAP samples using Trizol LS (Life Technologies) and a purification kit (Zymo Research) then quality-tested using RNA Pico Chips and BioAnalyzer 2100 (Agilent Technologies). All RIN values were >8.

#### RNAseq and TRAPseq

Libraries were prepared from 1 ug RNA using a library prep kit (NEB), rRNA depletion kit (NEB), and Ampure XP beads (Beckman), and following manufacturer’s instructions. Prior to sequencing, quality was tested using a High Sensitivity D1000 ScreenTape (Agilent Technologies). Each library was sequenced as 2 × 150 bp fragments to a depth of about 30 million reads using Illumina HiSeq3000 machines.

#### qPCR

PTZ and Sal treatment, brain harvest, and TRAP were performed as described above. Only mice with a 10-min seizure response were included. cDNA synthesis, reaction mixture setup, and qPCR were performed as per manufacturer’s instructions (QuantaBio #101414, Thermo # A25743, ABI QuantStudio 6Flex). Beta actin was used as a loading control. The primers used are described in Table S8.

#### Gene Ontologies and pathway analysis of translationally regulated genes

The BiNGO (Maere et al., 2005) tool within Cytoscape was used to define and plot enrichment of Gene Ontology categories within differentially expressed genes. The *Mus musculus* GOSlim_Generic annotation was used, inputting either the list of 102 PTZ upregulated or 315 PTZ downregulated (Table S3) astrocyte transcripts and displayed categories reaching 0.05 significance by hypergeometric test after Benjamini-Hochberg correction for multiple testing. This analysis was conducted once in an exploratory setting, using the whole genome as a reference, and a second time using a more conservative reference of the 3,410 astrocyte-enriched transcripts (Table S1), as highlighted in results. Full GO results are included in Table S5. The same gene lists were examined using the COMPBIO literature mining tool (https://gtac-compbio.wustl.edu/), with default parameters.

#### Comparison to curated gene lists

TRAP enriched transcripts were compared to prior markers of astrocytes as defined by a pSI <0.01 in a prior TRAP experiment (Dougherty et al., 2012), a pSI <0.01 of the immunopanned astrocyte expression (Zhang et al., 2014), analyzed as described (Ouwenga et al., 2017), and transcripts with a log2FC of >1, compared to nuclear transcriptomes of both neurons and oligodendrocytes (Reddy et al., 2017). TRAPseq upregulated and downregulated transcripts were further compared to the 102 genes identified by the TADA algorithm (Satterstrom et al., 2018) as causing ASD, and those genes curated as syndromic by the SFARI Gene database (Abrahams et al., 2013), accessed 8/9/19. Finally, they were also compared to locally translated transcripts in neurons (Ouwenga et al., 2017) or astrocytes (Sakers et al., 2017). All comparisons were conducted using a Fisher Exact Test, with the maximum number of genes set to the number measurably expressed in the current experiment (11,593).

#### Imaging based analysis of translation in astrocytes *in vivo*

We sparsely labeled astrocytes, as previously described (Sakers et al., 2017). Briefly, 2 μL of AAV9-CBA-IRES-GFP virus (concentration: 10^12^ vector genome (vg)/mL, obtained from the Hope Center Viral Vectors Core at Washington University) was injected at postnatal day 1–2 (P1-P2) pups bilaterally in the cortex, 1.5 mm lateral from the midline in two regions: 1 mm caudal to bregma and 2 mm rostral to lambda, using a 33 g needle (Hamilton #7803–05) with a 50 μL Hamilton syringe (#7655–01).

At P21, acute cortical slices (300 μm) were prepared in artificial cerebrospinal fluid (aCSF, in mM: 125 NaCl, 25 glucose, 25 NaHCO_3_, 2.5 KCl, 1.25 NaH_2_PO_4_, equilibrated with 95% oxygen, 5% CO_2_ plus 0.5 CaCl_2_, 3 MgCl_2_; 320 mOsmol) using a vibratome. 5 mM KCl, 5 mM KCl +1 μM tetrodotoxin (TTX) (Abcam), 50 ng/mL recombinant human/murine/rat BDNF (PeproTech, #450–02), or 10 mM PTZ were added to slices with 3 μM puromycin (Tocris #40-895-0) in aCSF and allowed to incubate for 10 min at 37°C. In the anisomycin condition, 1 mM anisomycin (Sigma #A9789) in aCSF was added to slices 30 min before puromycin at 37°C. Slices were fixed with 4% paraformaldehyde in PBS for 30 min, followed by 30 min in 30% sucrose and freezing in OCT (Sakura #4583) for cryosectioning.

We cryosectioned 40 μm sections into PBS and incubated with chicken anti-GFP (Aves, 1:1000), and mouse anti-puromycin (PMY) (Kerafast, 1:1000) at room temperature, followed by detection with appropriate Alexa conjugated secondary antibodies (Invitrogen), and counterstained nuclei with DAPI. We performed confocal microscopy on an AxioImager Z2 (Zeiss). Representative images represent 2–3 animals per condition with total cell number indicated in the figure legends.

#### Imaging based analysis of translation in astrocytes *in vitro*

Postnatal rat astrocytes were purified according to previously published immunopanning protocols (Foo et al., 2011; Li et al., 2019; Zhang et al., 2016). First, we coated separate immunopanning dishes (150 mm) with antibody against CD45 (BD 550539), Itgb5 (eBioscience), and a hybridoma supernatant against the O4 antigen (Zhang et al., 2016). Next, cerebral cortices were dissected from P3 rat pups and the tissue was digested into a single cell suspension using papain. We incubated dissociated cells sequentially on the CD45 and O4-coated panning dishes to remove microglia/macrophages and oligodendrocyte precursor cells. Astrocytes were isolated by incubating the single cell suspension on the Itgb5-coated panning dish. After washing away non-adherent cells, we lifted astrocytes bound to the Itgb5-coated dish using trypsin and plated them on poly-D-lysine coated plastic coverslips in a serum free medium containing Dulbecco’s Modified Eagle’s Medium (DMEM) (LifeTechnologies 11960069), Neurobasal medium (LifeTechnologies 21103049), sodium pyruvate (LifeTechnologies 11360070), SATO (Foo et al., 2011), glutamine (LifeTechnologies 25030081), N-acetyl cysteine (Sigma A8199) and HBEGF (Sigma E4643) on 24-well culture plates. We replaced half of the media with fresh media every 2–3 days.

AAV9-GFAP-LCK-CFP-MYC virus (concentration: 10^11^ vg/mL) was added to each well of rat astrocytes after 2 days *in vitro*. Medium was changed 24 h after infection, and we analyzed cells 5 days after infection to obtain ~30% sparse labeling prior to puromycylation. Astrocytes were treated with drugs in the same manner as acute slices with the following additions: KCl was tested at both 5 mM and 50 mM, TTX was tested at 1 μM with and without KCl, PTZ was tested at 10 mM, and untreated wells were used as controls. Duration of treatment was identical to acute slices methods, followed by two washes with fresh media and immediate 4% paraformaldehyde fixation.

Cells were permeabilized with 0.2% Triton X-100 and blocked with 10% donkey serum concurrently. Then we incubated coverslips with blocking solution, rabbit anti-GFAP (Dako, 1:1500), and mouse anti-PMY (Kerafast, 1:1000) overnight at 4°C, followed by detection with appropriate Alexa conjugated secondary antibodies (Invitrogen, 1:1000), and counterstained nuclei with DAPI. We acquired 5–10 images per well on a Zeiss Imager M2 microscope with an AxioCam MRm camera. Three independent experiments were conducted. Fluorescence intensity of PMY signal was quantified by drawing regions of interest (ROI) around individual cells using ImageJ (NIH) and analyzed using R as above.

#### Fear conditioning paradigm

Two cohorts of mice were tested in a fear conditioning paradigm described previously (Maloney et al., 2019; Nygaard et al., 2022). All animals were habituated to handling for multiple days prior to the start of testing. During testing, all males were run first, followed by females. All animals received subcutaneous injections of either 50 mg/kg CHX solution or Sal solution 30 min prior to the first day of conditioned fear testing. The first cohort of mice (n = 16 males, 16 females) underwent all three days of conditioned fear testing as previously described to assess the effect of CHX on fear recall. Testing occurred on three consecutive days, during which fear response was measured by quantifying percent of time freezing using FreezeFrame (Actimetrics) software. On the first day, animals were placed in the testing chamber, scented with mint extract, for five minutes. Freezing behavior was assessed during a two-minute baseline period and followed by three minutes of training during which a 2 s 1.0 mA foot shock was paired at the end of a 20 s 80 dB tone to condition a fear response to the tone and context. The second day tested contextual fear recall for eight minutes by placing the mouse in the same chamber but without the tone or shock. On the third day, the animal was placed in a new chamber with a novel scent (coconut) for unique context. During the first two minutes, baseline freezing to the new context was quantified, followed by eight minutes of the 80 dB tone to assess cued fear recall. One week after fear conditioning, animals were tested in a shock sensitivity protocol to assess reactivity to shock. Data were analyzed using SPSS (v27) and outliers or animals with unsuccessful injections were removed.

#### Synaptosome isolation

A second cohort of male C57BL/6Js were treated with cycloheximide (n = 9) or saline (n = 10), then underwent only the first day of fear conditioning, as described above. Following shock, they were sacrificed via cervical dislocation within four to six hours; the brain was removed, split in two, and the dorsal hippocampi were dissected, frozen on dry ice, then stored at −80°C until use. Synaptosomes were isolated on a discontinuous sucrose gradient as described previously (Gonzalez-Lozano et al., 2020). In brief, the dorsal hippocampus was homogenized in homogenisation buffer (0.32 M sucrose, 5 mM HEPES, in PBS. pH 7.4 with protease inhibitor cocktail (Roche)) using a Dounce homogenizer for 12 strokes at 900 rpm. After centrifugation at 1000 × g for 10 min, the supernatant was collected followed by centrifugation at 100,000 × g for 4 h in a 0.85/1.2 M sucrose gradient. Synaptosomes were collected from the interface, diluted, and centrifuged at 18,000 × g for 20 min to collect a synaptosome rich pellet. All steps were performed on ice or at 4°C settings.

#### FASP in-solution digestion of proteins

Filter-aided Sample preparation (FASP) was performed to digest the samples. 10 μg of synaptosomes from each sample was incubated with 75 μL reducing agent (2% SDS, 100 mM TRIS, 1.33 mM TCEP) at 55°C for 1 h at 900 rpm. Followed by incubation with MMTS for 15 min at room temperature. Next, samples were transferred to YM-30 filters (Microcon, Millipore) and 200 μL 8 M urea in 100 mM TRIS (pH 8.8) was added. Samples were washed with this solution five times by spinning at 14,000 × g for 10 min each time, followed by washing four times with 50 mM NH_4_HCO_3_. Finally, the samples were incubated with 100 μL of trypsin overnight in a humidified chamber at 37°C. Digested peptides were eluted from the filter with 0.1% acetic acid. The samples were dried using a SpeedVac and stored at −20°C.

### QUANTIFICATION AND STATISTICAL ANALYSIS

#### RNAseq and TRAPseq analysis

Sequencing results were quality-tested using FastQC (version 0.11.7). Illumina sequencing adaptors were removed using Trimmomatic (version 0.38) (Bolger et al., 2014), and reads aligning to the mouse rRNA were removed using bowtie2 (version 2.3.5) (Dobin et al., 2013). Surviving reads were then aligned, using STAR (version 2.7.0d) (Langmead and Salzberg, 2012), to the mouse transcriptome (Ensembl Release 97). The number of reads mapped to each feature was counted using htseq-count (version 0.9.1). All data are available on GEO: GSE147830.

Differential expression analysis was done using edgeR (version 3.24.3) (Robinson et al., 2010). Only genes with >5 CPM in at least 3 out of 12 samples were retained for further analysis (11,593 genes). A negative binomial generalized log-linear model (GLM) was fit to the counts for each gene. Then likelihood ratio tests (LRT) were conducted for comparing PTZ samples with Sal samples. The comparisons were listed as follow:

1. Ribosome Occupancy Changes = TRAPseq^PTZ^ - TRAPseq^Sal^
2. Transcriptional Changes = RNAseq^PTZ^ - RNAseq^Sal^
3. TRAP enrichment after PTZ = TRAPseq^PTZ^ - RNAseq^PTZ^
4. TRAP enrichment after Saline = TRAPseq^Sal^ - RNAseq^Sal^
5. TRAP enrichment, combined = (TRAPseq^PTZ^ + TRAPseq^Sal^)/2 - (RNAseq^PTZ^ + RNAseq^Sal^)/2

TRAP-enriched transcripts (Table S1) were defined as the union of all transcripts significantly enriched by TRAP (FDR <0.1) in the last three comparisons (#3–5). Transcriptionally altered transcripts (Table S2), were defined by having FDR <0.1 in comparison #2. Translationally altered astrocyte transcripts (Table S3) were defined as the intersect of Table S1 with the significant (FDR <0.1) transcripts from comparison #1, but removing those that were altered transcriptionally (i.e., also present in comparison #2). Results of all comparisons are included in Table S5. Code for these analyses is available (Sapkota et al., 2020).

#### qPCR

Fold changes in response to PTZ were calculated as 2^−DeltaDeltaCt^. A mixed linear model (DeltaCt ~ treatment +1|subject) was constructed, and the lme4 package was used in R to test DeltaCt as a function of PTZ treatment. p-value was calculated using a likelihood ratio test of the full mixed model with treatment against a model lacking treatment. Three animals per treatment were used.

#### Imaging based analysis of translation in astrocytes *in vivo*

Fluorescence intensity of PMY and GFP area were quantified using custom macros in ImageJ (NIH) and subsequently analyzed in R3.4.1 software. Data were analyzed by ANOVA using the car package in R and post-hoc pairwise t-tests with Bonferroni correction for multiple comparisons.

#### Mass spectrometry-based analysis

Samples were loaded onto an Ultimate 3000 LC system (Dionex, Thermo Scientific) as described (Gonzalez-Lozano et al., 2020; Hondius et al., 2021; van der Spek et al., 2021). A generally used hippocampal synaptosomes library created in-house with MaxQuant Software was used to annotate proteins. Spectra were annotated against the Uniprot mouse reference database.

Data quality control and statistical analysis were performed by using the downstream analysis 1024 pipeline for quantitative proteomics (Koopmans, 2020; MS-DAP version 0.2.6.3; for up to date version see https://github.com/ftwkoopmans/msdap). Outliers were removed when the variation among replicates was too large as observed by deviating distribution plots, or in the case of disturbed protein detection as observed by altered retention time plots. This led to a final sample size of n = 9 for the control (FC/CTL fear conditioning with saline) condition and n = 8 for CHX treated samples (FC/CHX fear conditioning with cycloheximide).

#### Mass spectrometry statistical data analysis

Peptide abundance values were normalized and the MSqRob algorithm was used for peptide-level statistical analysis. The threshold for significance was set at q < 0.05. For the Synaptic Gene Ontology database SynGO (1.1; (Koopmans et al., 2019)) analysis of synaptic proteins, all detected proteins were used as background input for analysis. Significance was determined by FDR correction. Cell type enrichment analysis was performed based on published proteomics data by (Sharma et al., 2015), where we annotated proteins to a certain cell type when the expression was > 2-fold higher for a specific cell type compared to the cell type with the second highest expression. Data visualisation and statistics were performed in R studio (RStudio Team (2020). RStudio: Integrated Development Environment for R. RStudio, PBC, Boston, MA URL http://www.rstudio.com/, version 1.3.1093) and GraphPad Prism (GraphPad Prism version 9.1.2. for Windows, GraphPad Software, San Diego, California USA, www.graphpad.com). Enriched gene lists were analyzed and visualized by BiNGO for molecular function enrichments, as described above, but using the set of all detectable proteins (Table S7) as the ‘background’ for the enrichment.

## Supplementary Material

1

2

3

4

5

6

7

8

## Figures and Tables

**Table:** KEY RESOURCES TABLE

REAGENT or RESOURCE	SOURCE	IDENTIFIER
Antibodies		
Chicken anti-GFP (1:1000)	Aves	GFP-1020; RRID: AB_10000240
Mouse anti-puromycin (1:1000)	Kerafast	3RH1; RRID: AB_2620162
Goat anti-Chicken, Alexa Fluor 488 (1:400)	Invitrogen	A-11039; RRID:AB_142924
Donkey anti-Mouse, Alexa Fluor 647 (1:400)	Invitrogen	A-31571; RRID: AB_162542
Rat anti-mouse CD45	BD Biosciences	550539; RRID: AB_2174426
Anti-integrin beta 5 monoclonal antibody	eBioscience	14-0497-82; RRID: AB_467288
Rabbit anti-GFAP (1:1500)	Dako	Z0334; RRID: AB_10013382
Chemicals, peptides, and recombinant proteins		
Pentylenetetrazol (PTZ)	Sigma Aldrich	P6500
Ampure XP beads	Beckman Coulter	#A63881
Recombinant human/murine/rat BDNF	PeproTech	#450–02
Puromycin	Tocris	#40-895-0
Anisomysin	Sigma Aldrich	#A9789
Tissue Tek OCT	Sakura	#4583
Dulbecco’s Modified Eagle’s Medium	LifeTechnologies	11960069
Neurobasal medium	LifeTechnologies	21103049
Sodium Pyruvate	LifeTechnologies	11360070
Glutamine	LifeTechnologies	25030081
N-acetyl cysteine	Sigma Aldrich	A8199
HBEGF	Sigma Aldrich	E4643
Tetrodotoxin	Abcam	#120055
Critical commercial assays		
RNA Clean and Concentrator Kit	Zymo Research	R1014
NEBNext Ultra II RNA Library Prep Kit for Illumina	New England BioLabs	NEB #7770
NEBNext rRNA Depletion Kit (Human/Mouse/Rat)	New England BioLabs	NEB #E6310
Deposited data		
Raw and analyzed data	This paper	GEO: GSE147830
Code for analysis	This paper	Bitbucket: https://bitbucket.org/jdlabteam/ptz_sal/src/master/RNASeq_analysis/ (10.5281/ZENODO.7054545)
Experimental models: Organisms/strains		
Mouse: C57BL/6J	Jackson Labs	Strain No. 000664
Mouse: B6.FVB-Tg(Aldh1L1-EGFP/Rpl10a)^JD130Htz^	Jackson Labs (Doyle et al., 2008)	Strain No. 030247
Oligonucleotides		
Primers for qPCR, see Table S8	This paper	
Recombinant DNA		
CBA-IRES-GFP	This Paper	Packaged into AAV9 by Hope Center Viral Vectors Core at Washington University
GFAP-LCK-CFP-MYC	Sakers et al., 2017	Packaged into AAV9 by Hope Center Viral Vectors Core at Washington University
Software and algorithms		
FastQC		Version 0.11.7
Trimmomatic	Bolger et al., 2014	Version 0.38
Bowtie2	Dobin et al., 2013	Version 2.3.5
STAR	Langmead and Salzber, 2012	Version 2.7.0d
Htseq-count		Version 0.9.1
edgeR	Robinson et al., 2010	Version 3.24.3
BiNGO Tool	Maere et al., 2005	Cytoscape
COMPBIO	GTAC at WUSTL	https://gtac-compbio.wustl.edu/
TADA algorithm	Satterstrom et al., 2018	
SFARI Gene database	Abrahams et al., 2013	
FreezeFrame	Actimetrics	
MS-DAP	Koopmans et al. *under review*	Version 0.2.6.3; GitHub: https://doi.org/10.5281/ZENODO.7054911
SynGO	Koopmans et al., 2019	1.1
RStudio	RStudio Team 2020	http://www.rstudio.com/, version 1.3.1093
GraphPad Prism	GraphPad Software	Version 9.1.2 www.graphpad.com

## Highlights

- Astrocytes have a programmed translational response to neuronal activity
- BDNF and K+, cues of neuronal activity, trigger this response
- This response requires the presence of neurons
- Post-learning changes in astrocytic protein levels at synapses require translation
